# Critical Advances for Democratizing Ultrasound Diagnostics in Human and Veterinary Medicine

**DOI:** 10.1146/annurev-bioeng-110222-095229

**Published:** 2024-06-20

**Authors:** Ahmed El Kaffas, Jenny M. Vo-Phamhi, John F. Griffin, Kenneth Hoyt

**Affiliations:** 1 Department of Radiology, School of Medicine, Stanford University, Stanford, California, USA; 2 Department of Large Animal Clinical Sciences, Texas A&M University, College Station, Texas, USA; 3 Department of Small Animal Clinical Sciences, Texas A&M University, College Station, Texas, USA; 4 Department of Biomedical Engineering, Texas A&M University, College Station, Texas, USA

**Keywords:** diagnostic imaging, global health, point-of-care ultrasound, ultrasound

## Abstract

The democratization of ultrasound imaging refers to the process of making ultrasound technology more accessible. Traditionally, ultrasound imaging has been predominately used in specialized medical facilities by trained professionals. Advancements in technology and changes in the health-care landscape have inspired efforts to broaden the availability of ultrasound imaging to various settings such as remote and resource-limited areas. In this review, we highlight several key factors that have contributed to the ongoing democratization of ultrasound imaging, including portable and handheld devices, recent advancements in technology, and training and education. Examples of diagnostic point-of-care ultrasound (POCUS) imaging used in emergency and critical care, gastroenterology, musculoskeletal applications, and other practices are provided for both human and veterinary medicine. Open challenges and the future of POCUS imaging are presented, including the emerging role of artificial intelligence in technology development.

## INTRODUCTION

The use of ultrasound imaging in both human and veterinary medicine is wide ranging and continues to expand worldwide. With the advancement of newer handheld ultrasound systems and devices, noninvasive ultrasound imaging can facilitate timely and appropriate management of patients or alleviate the need for other diagnostic tests. Using a technology termed point-of-care ultrasound (POCUS) imaging, procedures can now be performed without the need for a patient to be physically present in a radiology department (1). This allows for rapid bedside tests, especially in emergency medicine or ambulatory settings where a formal radiological investigation would inevitably delay any diagnosis. POCUS technologies are impacting not only resource-rich settings but also resource-limited global health care (2). We hypothesize that POCUS tools, augmented by mobile and artificial intelligence (AI) technologies, could eventually dominate primary care and enable several at-home imaging applications (3). This democratization, and the already rapid evolution of ultrasound imaging across the health-care industry, was notably accelerated by the recent pandemic caused by coronavirus disease 2019 (COVID-19) (4). Given the broad range of diagnostic applications of ultrasound, the advancement of ultrasound imaging in routine human and veterinary medicine is an exciting and evolving prospect as a complement to any physical examination (5). In this review, we first briefly discuss traditional medical ultrasound, major technological advances, and applications of POCUS imaging, followed by a discussion of open challenges and conclusions.

## MEDICAL ULTRASOUND

### Ultrasound Instrumentation

Modern ultrasound systems are highly sophisticated signal-processing machines (6). As in any complex system, there are many trade-offs in implementation due to performance requirements, physics, and cost. Actual implementations also vary considerably among ultrasound manufacturers and system types. These distinctions aside, any ultrasound system has three major components: an imaging transducer, a processing unit with controls, and a display for visual guidance. The transducer is arguably the most important part of an ultrasound system. It transmits a sound wave that propagates through tissue and receives the backscattered ultrasound signals that are generated before conversion to an electrical form for analysis by the processing unit. The active elements in a typical ultrasound transducer are small piezoelectric elements (7). When excited with a short, high-frequency electrical signal, piezoelectric elements mechanically vibrate and generate an ultrasound pulse. The dominant frequency of this ultrasound pulse is the same as the electrical signal used for excitation. The transducer bandwidth defines the range of frequencies that can be efficiently transmitted and received. The inherent trade-off between tissue resolution and penetration depth guides the choice of transducer frequency for any ultrasound imaging procedure.

The processing unit is another major hardware component of an ultrasound imaging system. This component is responsible for synchronizing the generation of sound waves and the reflected ultrasound measurements. The processing unit incorporates an ensemble of electronic circuits such as transmit–receive switches, noise amplifiers, and data converters and digital processing units. It also allows for control of the ultrasound system from the console, the touch-screen display, or a combination of both. Basic user controls include power or output setting, imaging depth, number of focal zones, and amplifier gains (image brightness). For decades, research has focused on reducing hardware volume in addition to signal- and image-processing complexity with the development of efficient ultrasound imaging systems that provide performance and image quality comparable with those of conventional, bulky ultrasound imaging systems (8). This was made possible in part by the evolution of computer technology, which has introduced more miniaturized hardware components with increased computational speed and reduced power demands.

**Figure 1:**
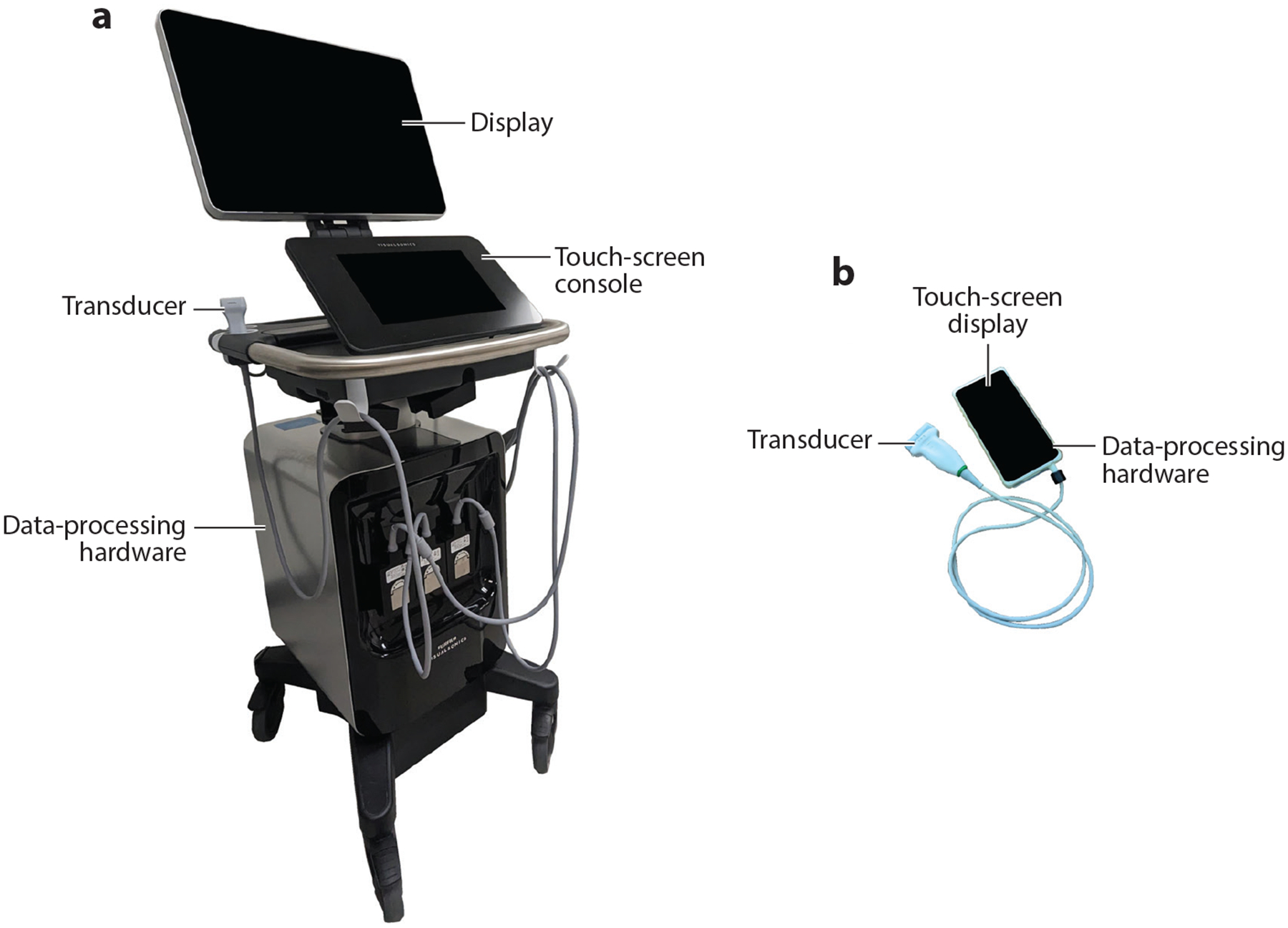
Examples of (*a*) cart-based and (*b*) handheld point-of-care ultrasound imaging systems.

Display technology is the last component of an ultrasound imaging system. This output device is responsible for the presentation of ultrasound images in real time after generation by the processing unit. A liquid crystal display (LCD) is a flat-panel device typically found on most modern ultrasound systems, but organic light-emitting diode (OLED) flat-panel displays represent a newer technology that provides increased image contrast (9). However, OLED displays are currently considerably more expensive than LCDs, which impedes their acceptance by ultrasound system manufacturers. In the case of POCUS, the display can be the touch screen of a small device such as a mobile phone. Collectively, progressive innovation in ultrasound and handheld transducer technology over the last decade has made portable POCUS imaging systems a reality. Examples of a cart-based and a handheld POCUS imaging system are presented in Figure 1.

### ALARA Principle

An acronym for “as low as reasonably achievable,” ALARA is an important concept in diagnostic ultrasound imaging (10). The ALARA principle assumes that any amount of exposure to ultrasound energy can increase (even minutely) the risk of adverse bioeffects. Within this framework, the guiding principle of ALARA is to keep patient exposure to ultrasound energy as low as possible for a given diagnostic result. To help inform these decisions, safety indices such as the mechanical index (MI) have been developed as an industry standard and are displayed in real time on ultrasound systems (11). The MI gives a relative indication of the potential for inducing a biological effect from tissue perturbation, which increases with higher MI levels. A prudent starting point for any ultrasound examination is to set the acoustic output to a low setting and then to modify it in a timely manner until the requisite diagnostic information is obtained. While the potential for ultrasound-induced biological effects exists if equipment is used imprudently (12), it is generally agreed that ultrasound exposure in typical clinical applications, including early obstetric imaging, has negligible risk and is very safe (13).

## TECHNOLOGICAL ADVANCES IN POINT-OF-CARE ULTRASOUND (POCUS)

From a technical viewpoint, POCUS offers the same major advantages as the more traditional ultrasound systems; however, POCUS systems tend to be offered at a lower cost with some compromise on features or image quality. POCUS devices are generally designed to offer increased versatility and portability, making them ideal for deployment in new clinical patient settings to improve access to care, provide rapid clinical decision support, and decrease delays in diagnosis. In this section, an overview of POCUS technologies and commercially available devices and their unique technological advantages will be highlighted.

### POCUS System Variants

The goal of POCUS is to provide imaging support to health-care practitioners beyond that provided by radiologists and cardiologists who typically have significant ultrasound experience or work with experienced sonographers to acquire images. Most POCUS systems are distributed by established ultrasound companies as a lower-end product that offers somewhat reduced quality images at a lower cost and provides greater convenience for the patient and provider. These systems are intended for applications at the point of care such as needle or procedure guidance or emergency triaging (e. g., fluid-accumulation-based and cardiac applications).

Cart-based POCUS systems were the first to emerge and have transducers made of traditional piezoelectric elements. An advantage of cart-based systems is that they interface with several transducer types that can be plugged into the cart system to optimize imaging for different applications. Personal computer–based systems allow for more advanced image processing and analysis and often support specialized software applications that provide diagnostic insights, automated calculations, streamlined documentation, and integration with hospital networks and electronic medical record systems. Tablet-based systems are highly portable, lightweight, and adaptable to various clinical settings including ambulatory, home-visit, and remote locations. Other advantages of tablet-based systems include fast start-up times, which make them especially valuable in time-sensitive situations, along with intuitive touch-screen interfaces, which make them user friendly and easy to navigate. More recently, a new class of handheld POCUS systems has emerged. These devices are highly portable and are augmented by the increased computing power available on personal computers and mobile smart devices (14, 15). Handheld POCUS systems can generally be interfaced directly with smart phones or tablets by either a wired (USB) or a wireless (WiFi/Bluetooth) connection.

### Transducer Arrays for POCUS

Several different piezoelectric transducer–based POCUS devices are commercially available, and each device offers varying degrees of image quality. This is often determined by the number and quality of piezoelectric elements in the transducer design, which can vary across ultrasound probes. Higher-density arrays with more elements allow for better spatial resolution. More elements can also provide greater flexibility in beam steering and focusing, resulting in improved image quality and diagnostic accuracy. Imaging speed and temporal resolution can be enhanced by multiline transmissions, where multiple elements transmit ultrasound waves simultaneously. Larger apertures with more elements allow for wider imaging coverage and deeper penetration.

More recently, ultrasound transducers fabricated using microelectromechanical systems technologies have been introduced (16). These transducer types use thin-film manufacturing principles to create arrays on a silicon substrate of small size and high sensitivity. Most notable is the Butterfly Network (Burlington, MA, USA), which offers capacitive micromachined ultrasound transducers (CMUTs) that use a series of tiny capacitive membranes to generate and receive ultrasound waves. Compared with piezoelectric transducers, CMUTs provide a broader bandwidth and allow for higher-frequency ultrasound imaging applications (17), although they require a high bias voltage to properly operate and may have long-term reliability issues (18). Elimination of manual assembly enables miniaturization during manufacturing and production of high-density CMUT arrays integrated with microelectronic circuitry (19). Similarly, piezoelectric micromachined ultrasound transducers (PMUTs) have been developed for POCUS imaging systems (20). Despite being fabricated with more challenging materials, PMUTs do not require a high bias voltage, which improves sensitivity in comparison with CMUT technology.

Piezoelectric-fiber composite transducers combine piezoelectric materials with optical fibers to offer greater flexibility, improved bandwidth, and compatibility with fiber optic sensing techniques (21). Optical ultrasound transducers generate and detect ultrasound waves using optical techniques (22). This phenomenon uses pulsed laser energy that is subsequently absorbed by biological tissues at depth and is followed by thermal elastic expansion, producing ultrasound waves that can then be acquired by a transducer to generate high-resolution images.

## APPLICATIONS OF POCUS

### Human Medicine

POCUS examinations differ from conventional ultrasound studies in that they are currently used to detect acute, potentially life-threatening conditions at bedside to expedite patient care. POCUS studies tend to be targeted to answer specific clinical questions that are instrumental in managing patients in real time. In contrast, comprehensive ultrasound examinations in radiology, obstetrics and gynecology (OBGYN), or cardiology settings carry out a complete ultrasound study of an anatomical region or organ system. Thus, a POCUS examination can take minutes to perform and interpret and relies on the use of lower-quality portable ultrasound systems. Conversely, a comprehensive exam is usually more time consuming and is performed by a dedicated operator or sonographer using higher-end ultrasound systems. Non-point-of-care medical imaging is established in radiology, OBGYN, and cardiology, but POCUS offers rapid real-time bedside image acquisition and interpretation that can be immediately integrated into the practice of patient-facing physicians. During vascular examinations, POCUS can help identify blood vessels and distinguish veins from arteries. POCUS can also support interventions and enable clinicians to rapidly triage or treat patients as needed, rather than wait for more extensive imaging. In OBGYN, POCUS is often used to rapidly detect changes in critically ill patients. Cardiac POCUS imaging can consist of a focused ultrasound examination and limited echocardiography (23). Vascular applications for POCUS include bedside assessment of lower-extremity deep vein thrombosis (DVT) (24) and screening for abdominal aortic aneurysm (25). Advanced POCUS systems are equipped with Doppler echocardiography, which can provide additional measures such as blood flow velocity and vascular congestion (26). Notwithstanding, POCUS comes with limitations, especially when only suboptimal experience or training is available to acquire or interpret the ultrasound images (27). Furthermore, POCUS still needs to be used together with other examination elements, such as inspection, palpation, and auscultation in cardiology (23). As discussed in more detail below, emerging technologies such as AI are rapidly being commercially deployed to provide support for new clinics adopting POCUS, and patients will benefit greatly from the incorporation of POCUS into the flow of examination procedures.

#### Emergency and critical care.

Emergency room (ER) physicians were among the first to adopt ultrasound into their clinical practice owing to the increased availability of POCUS systems. The advent of handheld POCUS has enabled increased use of ultrasound in the ER, with many clinicians using these devices like they do the age-old stethoscope. POCUS in an emergency setting can help diagnose conditions as varied as acute appendicitis, airway compromise, abdominal aortic aneurysm, and traumatic injury (28). Another diagnostic application that ER clinicians can use POCUS for is to rapidly identify DVT in lower extremities using vascular compression-based techniques (25, 29). Using POCUS and Doppler ultrasound imaging when available, ER physicians can also provide support to cardiologists for patients who present in the ER, through the detection of pericardial fluid or abnormalities in left-ventricular ejection fraction (28) or revascularization associated with occult occlusive myocardial infarction (30). In addition, POCUS can be used to guide interventional procedures that use deep needles (31). Emergency teams can also provide support to obstetrics teams through bedside emergency care, including rapidly confirming or dating a fetus in complicated pregnancies, identifying fetal heart activity, and assessing ectopic pregnancies (32). Finally, handheld POCUS was identified as an important tool for triaging and decreasing resource utilization during the COVID-19 pandemic (33) and has since been established for assessing pneumonia and pneumothorax (34). While the lung cannot be imaged with ultrasound, it is possible to identify lung consolidation through image artifacts that arise from fluid accumulation (35, 36). Other applications of POCUS in emergency and critical care generally include monitoring organ function and failure, confirming line placement, administering anesthetics, and detecting thromboembolic disease, among several others.

#### Musculoskeletal and sports medicine.

POCUS technologies that are portable (i.e., handheld) are ideal for musculoskeletal care, especially in the context of sports medicine where clinicians characterize injuries and support athletes at courtside or literally on the court. In general, POCUS for musculoskeletal applications has grown significantly in recent years. Podiatrists have adopted POCUS in private offices with a growing user base (37). POCUS has been used to rapidly identify muscle and tendon injuries, soft-tissue infections, long-bone fractures, joint dislocations, and effusions (38). POCUS can also help reduce the need for multiple radiographs to diagnose and manage shoulder dislocations (39). Use of ultrasound during reduction of a dislocation can eliminate the need for additional sedation and could potentially decrease ER admission times.

#### Gastroenterology.

Gastroenterology physicians have been using ultrasound for decades. In Europe, many gastroenterology physicians do their own ultrasounds for tissue imaging without relying on radiologists, focusing on abdominal organs such as the pancreas and liver. In the United States, ultrasound systems with endoscopic transducers are used extensively for image-guided fine-needle aspiration or biopsy of pancreatic tissue (40). Intracavitary contrast-enhanced POCUS can also aid in the ultrasound-guided management of abdominal fluid abscesses; however, contrast-enhanced ultrasound is not yet mainstream in POCUS applications and tends to be limited mainly to radiology or cardiology applications (41). With nonalcoholic fatty liver disease increasingly becoming a global health concern, systematic screening strategies are needed. Preliminary reports indicate that POCUS imaging and dedicated software solutions may help in the diagnosis and grading of early liver disease (42), which is a disease state that is reversible through diet and lifestyle changes. Hepatologists also routinely use a point-of-care, imageless, ultrasound-based device called FibroScan^®^ (Echosens, Paris, France) that remotely measures tissue stiffness for the assessment of advanced liver fibrosis or cirrhosis (43). Newer FibroScan systems with a measurement functionality that is sensitive to fatty liver now allow the noninvasive evaluation of steatosis (44).

#### Urology.

Urology is a top specialty that utilizes POCUS. These uses include applications in bladder, kidney, and prostate imaging. POCUS can be used to measure bladder volumes and identify kidney stones (nephrolithiasis) (45). Venous excess Doppler ultrasound (VExUS) of kidneys has been shown to aid in clinical decision-making (46). For the prostate, urologists use cart-based POCUS systems with transrectal probes to examine and perform image-guided biopsies for the diagnosis and staging of prostate cancer. There are also several reports of urologists outside of the United States using contrast-enhanced ultrasound in prostate and kidney applications with POCUS devices (47). The recent introduction of wireless handheld POCUS systems, including endocavity probes for transrectal or transvaginal imaging, is enabling new avenues in prostate care where urologists can use these devices within their offices during a regular visit. Other areas of interest in urology include undifferentiated acute scrotum assessment, renal colic evaluation, and guidance of suprapubic catheter placement (48).

#### Endocrinology.

POCUS is widely used in endocrinology. For example, ultrasound is the first-line imaging examination to assess the malignancy risk of thyroid nodules (49). POCUS is also used to guide procedures, biopsies, and surgeries. Ultrasound-guided percutaneous laser ablation is a minimally invasive and effective outpatient treatment for thyroid nodules. It is well tolerated, repeatable, and associated with a low major complication risk (50).

#### Pulmonology.

Lung ultrasound usage has increased in point-of-care settings more generally due to the wide range of clinically valuable assessments. The quantitative evaluation of a B-line score has become a useful POCUS tool in assessing pulmonary congestion (51). B lines are persistent hyperechoic artifacts that originate from the pleural surface and extend to the bottom of the ultrasound display screen (52). These features correlate with lung sliding and can provide an accurate measurement of pulmonary congestion, surpassing physical examination and chest X-ray procedures (53). Point-of-care, contrast-enhanced lung ultrasound can help visualize areas of small consolidations, a frequent sign of pneumonia (54).

#### Primary care.

Many primary care settings lack in-house radiology equipment. POCUS helps fill this void by offering immediate, cost-effective access to imaging, enabling physicians to obtain diagnostic information without referrals or waiting for external imaging studies (55). There are numerous reasons to use ultrasound in an outpatient family medicine setting including screening for abdominal aortic aneurysm, confirming intrauterine pregnancy and fetal position, assessing left ventricular function, identifying cholelithiasis, and guiding joint injections. These have all been studied and are practical uses for many family physicians (56). Family medicine residency programs are now increasingly including POCUS training in their curriculum (57).

#### At-home care.

The portable and user-friendly nature of POCUS imaging makes it well suited for at-home care (58). Patients can easily perform ultrasound examinations with in-person or remote guidance from health-care providers. The portability of POCUS allows for imaging at the patient’s location, eliminating the need for inconvenient and time-consuming transportation to medical facilities. Notwithstanding, the accuracy of POCUS depends on the skills and judgment of the operator (3). While there is growing interest in this area, at-home care with handheld POCUS systems remains uncommon.

#### Low-resource settings.

POCUS has significant potential to improve access to imaging in low-resource settings, especially for obstetric, disease, or trauma-related indications (59). Where other human medical imaging modalities are scarce, handheld POCUS specifically offers a versatile, pocket-sized solution. Increased affordability and awareness of POCUS imaging are reducing the barriers to its implementation in rural settings, conflict zones, and disaster sites (60).

### Veterinary Medicine

Nearly two decades ago, it was established that a focused assessment with sonography for trauma (FAST) could detect free abdominal fluid in dogs following motor vehicle accidents and blunt force injury (61). Importantly, this landmark study demonstrated that POCUS was a simple and rapid technique that could be performed on animals in an emergency setting by veterinary clinicians with minimal ultrasound imaging experience. This was reinforced by a more recent study that found that at least 20 scans were needed before a veterinarian could perform FAST examinations in a repeatable and proficient manner (62). Today, veterinarian specialists and general practitioners across the world have incorporated POCUS for performing fast and focused patient examinations to answer an array of diagnostic questions or guide performance of invasive procedures (63, 64).

#### Abdominal POCUS.

In additional to identifying the presence of free fluid in emergency cases, POCUS provides visualization of abdominal organs and soft tissues. For example, POCUS evaluation of the liver can be used to localize solidary hepatic masses (65) and local recurrence after surgical excision (66). In patients suspected of having neoplastic processes, POCUS can rapidly stage the patient for localized versus disseminated disease (67). In cancer patients, ultrasound can monitor tumor size and guide serial biopsy or fine-needle aspiration during therapy. A hepatobiliary POCUS examination can also detect various liver diseases with structural changes such as chronic hepatitis and fibrosis (68). Detection of hepatic vascular anomalies without the use of general anesthesia is also possible (69). POCUS of the liver and gallbladder can help guide clinical decisions by providing initial information that may not be evident from review of abdominal radiographs. In horses with signs of acute abdominal pain, ultrasound can be used to detect intestinal obstruction (70).

#### Thoracic POCUS.

Details of the thoracic FAST examination were first published in 2008 (71). This seminal clinical study focused on the diagnosis of pneumothorax (collapsed lung) in dogs with blunt and penetrating trauma. Today, POCUS is routinely used in the emergency room to quickly detect pneumothorax in traumatized dogs and cats with high sensitivity and specificity, as well as assessing the degree of pneumothorax to help determine clinical significance (72). POCUS can also diagnose and monitor pleural and pericardial effusion. In addition to providing valuable information on basic heart function and pathology, cardiac POCUS imaging can be used for the rapid assessment of congestive heart failure (73) or responsiveness to intravenous fluids as an essential component of shock management to increase cardiac output and improve tissue perfusion (74).

#### Musculoskeletal and neurological applications.

Most frequently caused by road traffic accidents, traumatic brain injury is a common occurrence in veterinary medicine and results in a high rate of morbidity and mortality in small animals (75). Successful outcomes depend upon the severity and location of injury, appropriate management, and timely identification of complications. While advanced imaging is the reference standard for diagnosing traumatic brain injury, POCUS imaging is increasingly being used to detect structural lesions and assess vascular perfusion (76). Musculoskeletal ultrasound is another established field in veterinary medicine. An advantage to using POCUS in musculoskeletal applications is that the oppositive limb is commonly used for comparison to evaluate the symmetry of structures such as tendons and ligaments (77).

#### Reproductive and pediatric applications.

While most domesticated pets have undergone surgical sterilization, ultrasound imaging can be used to confirm a patient’s reproductive status, since many adopted dogs and cats have an unknown medical history. In small animals, POCUS can help determine causes of scrotal swelling and be used to screen for pathology of the prostate in male patients. With female small animal patients, reproductive POCUS can be used to diagnose pregnancy and life-threatening infection in the uterus. Uses of real-time POCUS imaging in reproductive health management of horses, cattle, and other large domestic species have also been well documented (78). For example, use of a POCUS system equipped with a transrectal imaging transducer allows reproductive examinations of cattle by a bovine practitioner, which represents a considerable advance to the dairy industry (79). On-farm use of ultrasound imaging in young cattle has emerged as a practical tool for identifying lung lesions associated with bovine respiratory disease (80). POCUS is also commonly used for imaging the abdominal cavity and for the detection of any gastrointestinal obstruction or perforation from a foreign body, as well as for the assessment of urogenital disorders and to assess vascular and digestive system development (81). POCUS examinations of animals that are noncompliant, aggressive, tense, panting, excessively large, or obese tend to be more challenging. Sedation is often used to improve scan quality.

#### Vascular examinations.

During vascular studies, Doppler ultrasound technologies can be used to identify blood flow and direction. POCUS can also screen for evidence of vascular thrombosis and other conditions associated with adverse hemodynamic changes due to venous congestion (82) or arteriovenous malformation (83). When combined with standard ultrasound image findings such as echogenicity (brightness) and organ anatomy, vascular POCUS can guide diagnostics and therapy for conditions that alter parenchymal perfusion due to acute kidney injury (84) and chronic kidney disease (85). In emergency situations, a rapid vascular access can be a lifesaving procedure for the administration of sedatives, analgesics, anesthetics, and drugs. To that end, ultrasound guidance during these catheterizations can improve the success rate and reduce any potential complications (86). These ultrasound-guided vascular access techniques are growing in acceptance with the increasing availability of POCUS systems in veterinary practices.

More recent technical developments have enabled high-resolution vascular imaging using contrast-enhanced ultrasound (87, 88). After bolus injection of an intravascular microbubble agent with a circulatory half-life on the scale of minutes (89), real-time, contrast-enhanced POCUS verifies tissue perfusion and has aided in the diagnosis of numerous diseases of the bladder, kidney, liver, pancreas, spleen, and lymph nodes (90, 91). For example, by evaluation of hemodynamic (image enhancement) patterns, contrast-enhanced POCUS imaging can help identify hepatic arterioportal fistulas (92). Initial contrast-enhanced POCUS studies also demonstrated improved visualization of tumorous tissue (93) and could distinguish between benign and malignant lesions (94, 95). Given the numerous studies that have detailed a very low incidence of adverse events and the safe use of contrast-enhanced POCUS in animal patients (96–98), many more diagnostic applications in veterinary medicine could follow. Notwithstanding, while contrast-enhanced ultrasound is a promising technique for POCUS applications, more research into diagnostic accuracy is warranted to prove value in both human and veterinary medicine.

## OPEN CHALLENGES AND THE FUTURE OF ULTRASOUND

### Training and Standardization of Protocols

The adoption of ultrasound technology by nontraditional users has been rapid and far reaching. In many countries, health practitioners may perform ultrasound procedures with little or no training or formal accreditation, leading many to call for reform and more regulation of ultrasound use (99). More pointedly, POCUS remains largely unregulated globally (100). Demands for a standardized curriculum and longitudinal competency assessment are motivated in part because ultrasound is a highly user dependent modality and because imaging examinations performed by untrained health practitioners may represent a higher risk of misdiagnoses (101). While accurately conducting ultrasound imaging procedures is a teachable skill, focused training, practice guidelines, and standardization of image acquisition protocols are required to meet the needs of new users and patients (102). The standardization of all ultrasound image acquisitions, guided by ALARA principles, as well as the interpretation of protocols, is critically important to ensure correct diagnoses and to avoid unnecessary tests and inappropriate treatments. Any training process would certainly vary depending on prior experience and ultrasound user interests.

Radiologists and ultrasound technicians undergo a structured training program that takes years to complete (103). As a requirement of employment by many health-care providers, maintenance of certification helps demonstrate support for continuous quality improvement, professional development, and delivery of quality patient care. Unlike comprehensive ultrasound services, POCUS examinations are more targeted studies used to achieve specific procedural aims or to answer focused questions. Therefore, formal needs-based training programs for POCUS users may not be as extensive (or standardized) as the more advanced programs in ultrasound that are required for those performing comprehensive ultrasound imaging procedures (104). While numerous POCUS user training and assessment programs exist, they are in their infancy, and there is no consensus framework so the quality and value of each can differ. In general, a comprehensive POCUS training program might include a collection of online learning modules to strengthen and refine ultrasound knowledge. In-person courses would then provide a review of key elements of POCUS applications and the ability to practice quality image acquisition on human or animal models under the guidance of skilled ultrasound users. Longitudinal attendance at approved regional ultrasound courses would allow POCUS users to reinforce and refine the skills necessary to ensure continued delivery of quality patient care. At some point in the training program, it would be critical to educate users on how to properly report POCUS examination results and ensure they are included as part of the patient’s clinical record. Overall, evaluation of POCUS user competency could be evaluated by a comprehensive skills and knowledge assessment as well as formal certification (105). As more formal POCUS training programs become available, future research should gauge the diagnostic accuracy of ultrasound and the impact on patient outcomes and utilization in different resource-rich and resource-limited global health-care settings.

### Equipment Maintenance and Service

Regular maintenance and reliable equipment servicing are critically important steps to ensure that diagnostic ultrasound imaging systems continue to function properly and retain usefulness (asset value). In many hospitals and clinics, facility accreditation requires that all ultrasound equipment be fully inspected by a trained professional to ensure they meet established guidelines and undergo a routine preventive plan (calibration) to ensure quality control (106). In the United Sates, this accreditation is provided by renowned nonprofit organizations such as the American Institute of Ultrasound in Medicine and the American College of Radiology. While the programs are voluntary, imaging facilities with accreditation have advantages over nonaccredited facilities, including prestige and access to procedure reimbursements from some state agencies and private insurance companies. Given that POCUS has become increasingly common in locations remote from conventional health-care facilities, comparable incentives for POCUS system maintenance and servicing are inadequate or nonexistent. In resource-limited health-care settings, repair costs and restricted access to servicing professionals may place additional barriers on having a properly functioning POCUS system. Notwithstanding, access to routine POCUS system testing, calibration, and repair is critically important to ensure accurate patient diagnoses. When regional service providers are unavailable, cost effective and timely online and package delivery solutions should become ubiquitous.

### Emerging Role of Artificial Intelligence in POCUS

The use of POCUS technology (especially handheld POCUS) is exploding, with new clinical users adopting these technologies for health-care delivery. Nonetheless, many new users are not trained in acquiring or interpreting ultrasound images. This is especially true in complex imaging scenarios. To help address this shortcoming, AI has been proposed and demonstrated as a tool to augment POCUS operation by providing users with assisted image acquisition and interpretation. Both academics and entrepreneurs have enabled innovative AI solutions to support POCUS use.

Cardiology is one specialty that has undergone significant improvements in POCUS thanks to AI. For example, AI models have been developed to provide acquisition guidance in visualizing specific views of the heart (107). Deep learning (DL) models trained on hand movements of skilled cardiac sonographers have successfully been used in a COVID-19 intensive care unit to guide inexperienced users in acquiring high-quality, bedside, cardiac ultrasound images (108). More specifically, a sizable group of critical care trainees with limited experience used an automation-assisted technique that provided real-time feedback to help acquire better POCUS images than were previously possible. This consequently allowed more accurate measurement of aortic outflow velocity as well as enabling quick and reliable assessment of cardiac output variations and other hemodynamic effects of fluid resuscitation in a setting such as shock management. More recently, POCUS with the assistance of ChatGPT (OpenAI, San Francisco, CA, USA), which is a large language model, has been used to diagnose and document ventricular septal rupture following acute myocardial infarction (109).

Gains in AI-assisted POCUS are also being made in pulmonology. The previously mentioned B-line score technique for assessing pulmonary congestion using POCUS has not achieved widespread usage, partly because the assessment is tedious. Addressing this, DL models using convolutional neural networks have been developed to automatically quantify B-line scores from POCUS lung videos (110).

Most existing AI algorithms developed for ultrasound applications were trained on datasets captured from retrospective studies. A few commercially developed models are the exception. Ongoing research typically aims to develop DL models for ultrasound that can be adapted for POCUS devices. With limited computing power, integration of AI software on existing ultrasound hardware poses a near-term challenge. Federal approval can occur once AI-based POCUS systems have been clinically validated and demonstrated to be safe and effective. Similar regulatory milestones have been achieved in other medical imaging applications. In 2018, the US Food and Drug Administration approved a pioneering retinal imaging device with onboard AI that can make important diagnostic decisions (111). Achievements like this, and others, help highlight the potential for AI-integrated medical imaging solutions including POCUS technologies.

## CONCLUSIONS

The role of ultrasound in medical diagnosis and patient management continues to evolve. Ultrasound allows medical imaging to impact not only radiology but also other specialized areas of medicine. This democratization of ultrasound is driven in part by new technological advancements, applications, and users. As portable ultrasound systems become more ubiquitous, acquisition and interpretation of images can be performed at the point of care, allowing trained users to reach a rapid and reliable diagnosis that can help guide patient management.
